# Vitamin D levels in children and adolescents are associated with coronavirus disease-2019 outcomes: A systematic review and meta-analysis

**DOI:** 10.1097/MD.0000000000040245

**Published:** 2024-11-01

**Authors:** Ji-Gan Wang, Hui-Hong Dou, Qiong-You Liang

**Affiliations:** a Department of Pediatrics, Maternal and Child Health Hospital of Guangxi Zhuang Autonomous Region, Guangxi Clinical Research Center for Pediatric Diseases, Nanning, 530003, China.

**Keywords:** COVID-19, children, meta-analysis, SARS-CoV-2, vitamin D

## Abstract

**Background:**

To explore the relationship between vitamin D levels and risk of SARS-CoV-2 infection and its severity in children and adolescents, and provide a theoretical basis for clinical practice.

**Methods:**

The PubMed, Web of Science, Embase, MEDLINE, and Cochrane Library databases were searched for comprehensive cohort and case–control studies on the association between childhood vitamin D deficiency and the risk and prognosis of coronavirus disease-2019 (COVID-19). The search period was set from December 1, 2019, to December 31, 2023.

**Results:**

The vitamin D insufficiency rate in children with COVID-19 was 80.78% (95% CI, 62.6% to 93.89%), with a deficiency rate of 32% (95% CI: 19.01% to 46.61%). Vitamin D insufficiency was more common in children with COVID-19 than in healthy children (OR, 4.86; 95% CI: 2.56–9.26). The incidence of severe illness was higher (OR, 4.73; 95% CI: 1.39–16.11) whereas that of asymptomatic illness was lower (OR, 0.38; 95% CI: 0.38–0.81) in children with COVID-19 who had vitamin D insufficiency than in those who did not.

**Conclusions:**

Vitamin D insufficiency in children may increase the risk of COVID-19 infection and is associated with poor prognostic outcomes. Further research is required to confirm the optimal Vitamin D dose to prevent insufficiency in various sections of the population.

## 1. Introduction

Although coronavirus disease-2019 (COVID-19) has been prevalent for 3 years, and different vaccines have been developed, treatment outcomes are still not satisfactory.^[1,2]^ Currently, no specific drugs for treatment of the disease exist; therefore, symptomatic treatment is generally followed. Vitamin D regulates systemic inflammatory responses by interacting with most cells of the immune system, meaning that it may have a protective effect against respiratory infections and other diseases.^[3]^ Moreover, it acts on adaptive immunity by modulating pro-inflammatory cytokines, including interleukin (IL)-6, tumor necrosis factor (TNF)-alpha, and interferon-gamma, and controlling the response mediated by Th1 lymphocytes.^[4]^ This regulation is expected to be less efficient in cases of vitamin D deficiency, though it can be restored after adequate supplementation; therefore, vitamin supplementation may have a preventive effect against COVID-19. Over the past 3 years, several researchers have investigated the role of vitamin D in the pathophysiology of COVID-19, and studies have shown a positive association between vitamin D deficiency and COVID-19 severity.^[5,6]^ Nevertheless, a meta-analysis found that vitamin D supplementation did not significantly affect COVID-19 outcomes.^[7]^ With the easing of the epidemic prevention policy, emergence of new variants, and disparities in the efficacies of novel drugs, children must be protected from COVID-19 while not being subject to novel drugs. Vitamin D deficiency may be an easily modifiable risk factor, especially given the low cost and safety of vitamin D supplements. Therefore, this systematic review and meta-analysis aimed to understand the association between vitamin D supplementation and susceptibility to and outcomes of COVID-19 in children, and provide new references for prevention and treatment.

## 2. Methods

### 2.1. Definition

The vitamin D status was considered sufficient at a serum 25(OH)D level of at least 30 ng/mL (75 nmol/L), insufficient at a level of 21 to 29 ng/mL (52.5–72.5 nmol/L), and deficient at a level of < 20 ng/mL (<50 nmol/L).^[8]^

COVID-19 severity was defined based on clinical characteristics, laboratory testing, and chest imaging, including asymptomatic infection, mild, moderate, severe and critical cases (National Health Commission of People’s Republic of China, Diagnostic and treatment plan of Coronavirus disease-2019 (7th ed.) (2020)).

### 2.2. Literature search

This systematic review was conducted according to a prospectively registered protocol (CRD42023394711). This study is reported in accordance with the 2009 guidelines of the Preferred Reporting Items for Systematic Reviews and Meta-analyses (PRISMA) statement (PRISMA 2009 Checklist, Supplemental Digital Content, http://links.lww.com/MD/N788). The PubMed, Web of Science, Embase, MEDLINE, and Cochrane library databases were searched both online and manually, and references of the included literature were traced. We used a combination of subject terms and free words, adjusted according to the characteristics of different databases.

The PubMed retrieval strategy was as follows: ((((Vitamin D) OR (VD)) OR (1,25(OH)2D)) OR (VitD)) AND ((((2019-nCoV) OR (COVID-19)) OR (SARS-CoV-2)) OR (Corona Virus Disease 2019)))) AND ((((Pediatrics) OR (Pediatrics)) OR (children)) OR (child)).

Other database search strategies can be found in the Search Strategy, Supplemental Digital Content, http://links.lww.com/MD/N788).

### 2.3. Literature screening and data extraction

The literature was searched and screened independently by 2 researchers, and the data were collected and cross-checked. Disputes, if any, were resolved through discussion or consultation with a third researcher.

The inclusion criteria were as follows: 1) Research type: cohort study, case–control study and case series. 2) Study object: children diagnosed with COVID-19. 3) Observation indicators: Relationship between vitamin D deficiency and risk and prognosis of COVID-19 (incidence rate and state of illness).

The exclusion criteria were as follows: 1) Repeated publications of the same study; 2) Short case reports; and 3) Missing or incomplete analysis data or data that could not be obtained even after contacting the author.

### 2.4. Assessment of risk of bias in the included studies

The Newcastle-Ottawa quality assessment scale for case–control studies was adopted to assess the quality of included studies.^[9]^ This scale has 3 categories and 8 items. Two researchers performed quality assessments individually, with assessment values ranging from 0 to 9 stars. Each band indicates the percentage of included studies meeting each of the quality criteria. The following 3 aspects were evaluated: selection of participants, comparability between groups, and outcome or exposure factor measurement. Studies with a total score < 4 were classified as low-quality, those with a score of 4 to 6 as medium-quality, and those with a score > 6 as high-quality.

### 2.5. Statistics

Meta-analysis was performed using R version 3.6.3 software. Odds ratios (ORs) and 95% confidence intervals (CIs) were used to calculate the effect size for dichotomous variables. A meta-analysis of incidence of vitamin D deficiency/insufficiency in patients with COVID-19 was performed, and the combined incidence was calculated with a 95% confidence interval (CI). The heterogeneity was assessed using I^2^ and Q statistics, and an I^2^ > 50% was considered to indicate heterogeneity among studies. The data were analyzed using a random effects model.

### 2.6. Patient and public involvement

Patients and/or the public were not involved in this research’s design, conduct, reporting or dissemination plans.

## 3. Results

### 3.1. Literature screening process and results

**Figure 1.**
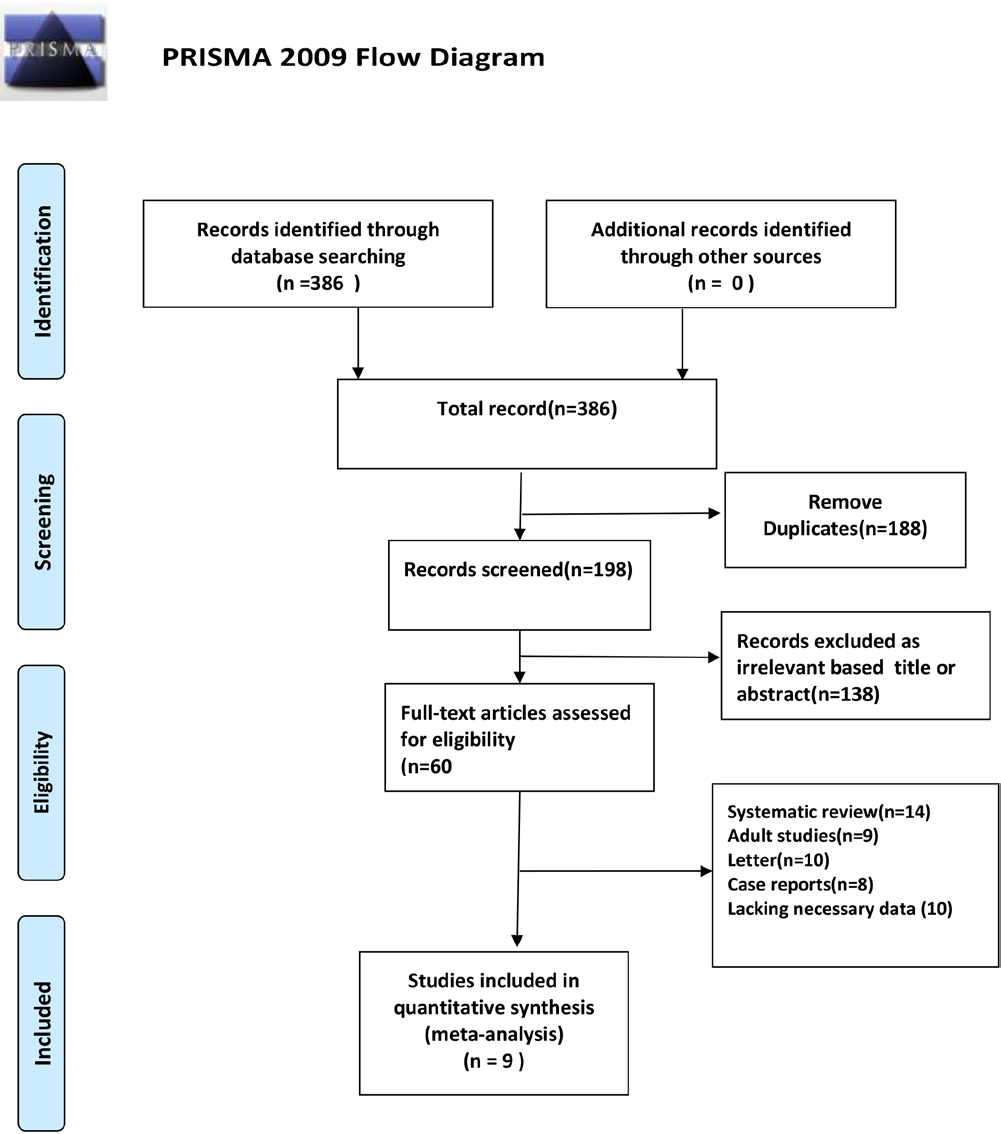
Flow diagram for identification of selected studies in the meta-analysis.

The flow diagram shows the detailed steps of the literature search (Fig. 1). A total of 386 related studies were obtained upon initial examination, 188 duplicate studies were removed, and 138 studies were removed after reading their titles and abstracts. Finally, after layer-by-layer screening, a total of 9 studies comprising 1262 children were included.^[10–18]^ The basic characteristics of included studies are presented in Table S1, Supplemental Digital Content, http://links.lww.com/MD/N788).

### 3.2. Basic characteristics and quality evaluation results of included studies

The quality scores of the included studies ranged from 4 to 8, indicating that all of them were medium-to-high quality studies (≥4 points; Table S2, Supplemental Digital Content, http://links.lww.com/MD/N788).

### 3.3. Meta-analysis results

#### 3.3.1. Prevalence of vitamin D deficiency in children with COVID-19

**Figure 2.**
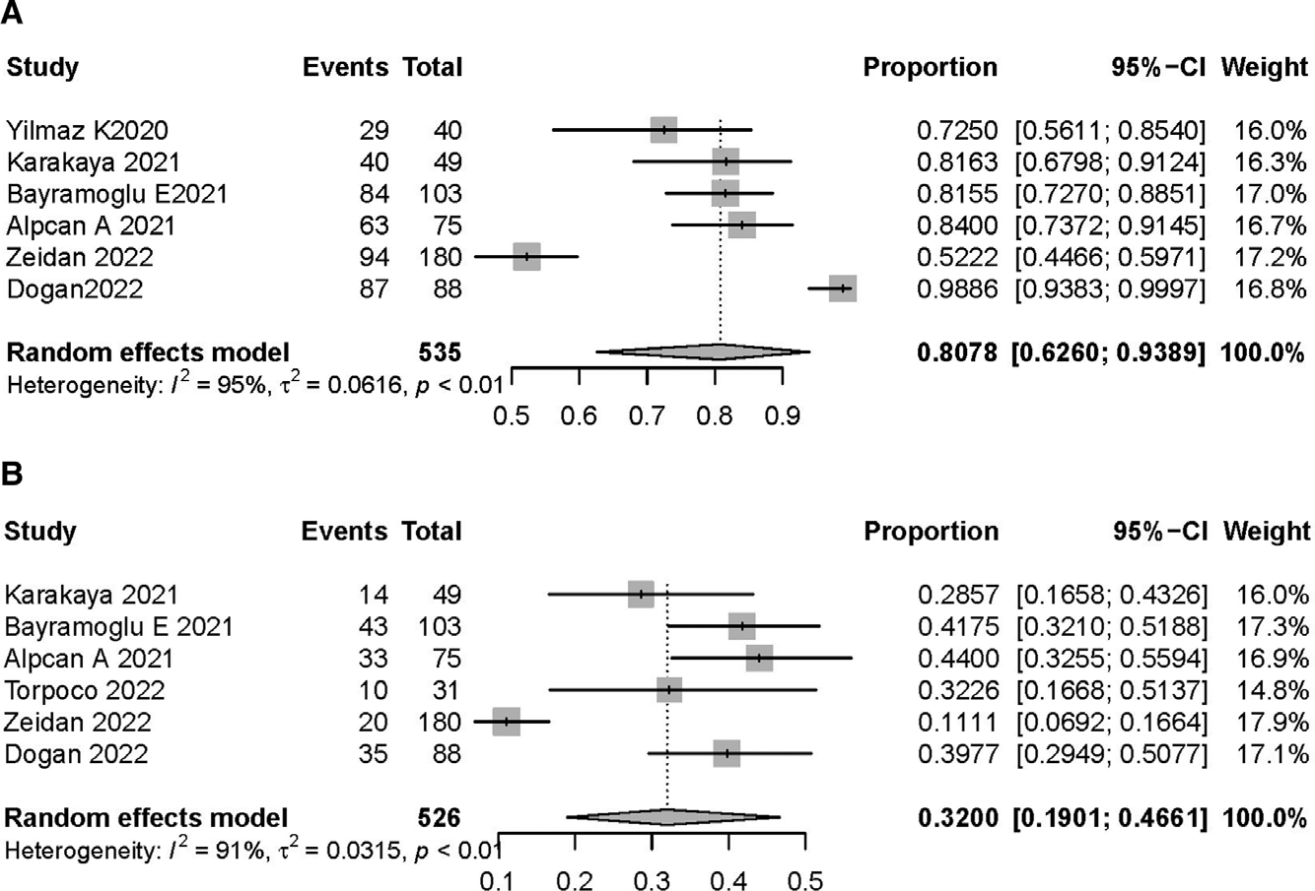
Forest plot the single-arm meta-analysis of the incidence of vitamin D insufficient and deficiency.

Six studies^[10–13,15,18]^ comprising 535 children with COVID-19 reported vitamin D deficiency rates. Owing to high heterogeneity (*I*^2^, 95%), a random effects model was adopted, and meta-analysis results showed that the vitamin D insufficiency rate in children with COVID-19 was 80.78% (95% CI, 62.6%–93.89%; Fig. 2A), with a corresponding deficiency rate of 32% (95% CI, 19.01%–46.61%; Fig. 2B).

#### 3.3.2. Association between vitamin D insufficiency and risk of COVID-19

**Figure 3.**
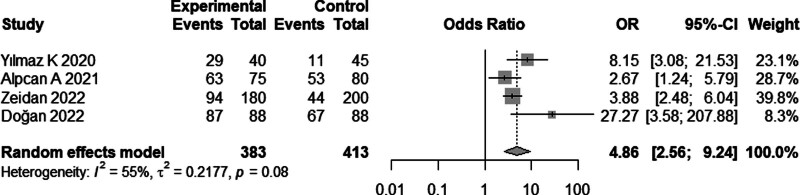
Forest plot of the 2-arm comparison (meta-analysis B) of incidence of vitamin D deficiency in children with COVID-19 and healthy children.

**Figure 4.**
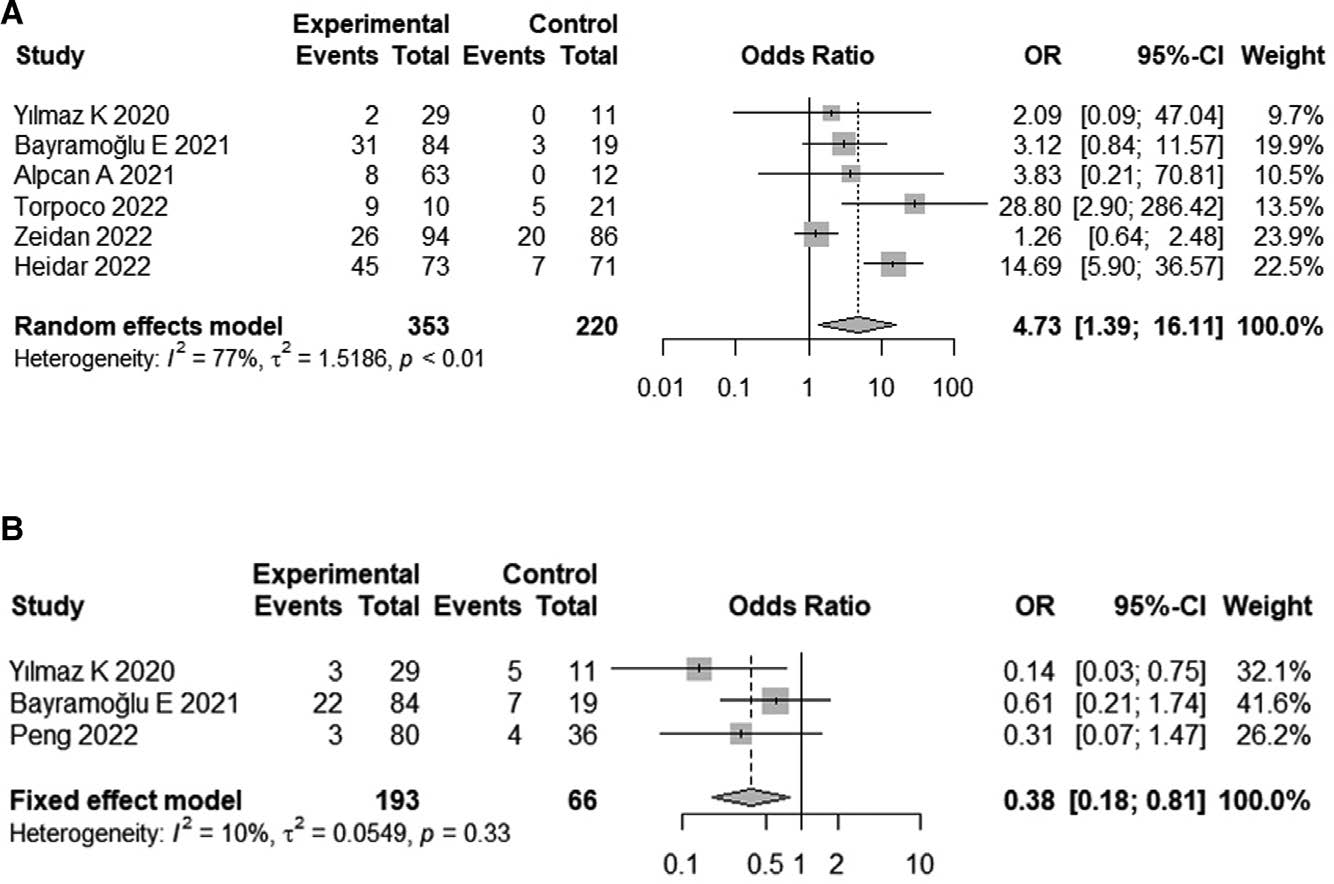
Forest plot of comparing clinical manifestations of disease in vitamin D deficient and vitamin D normal groups.

Four studies^[10,13,15,18]^ comprising 796 children reported the incidence of vitamin D insufficiency in both children with COVID-19 and healthy children. Meta-analysis results suggested that vitamin D insufficiency was more common in children with COVID-19 than in healthy children (OR, 4.86; 95% CI: 2.56–9.26; Fig. 3). Among children with COVID-19, the vitamin D insufficiency group had a higher incidence of severe infection (OR, 4.73; 95% CI: 1.39–16.11; Fig. 4A) and a lower incidence of asymptomatic disease (OR, 0.38; 95% CI: 0.38–0.81) than the normal vitamin D group (Fig. 4B).

### 3.4. Publication bias

**Figure 5.**
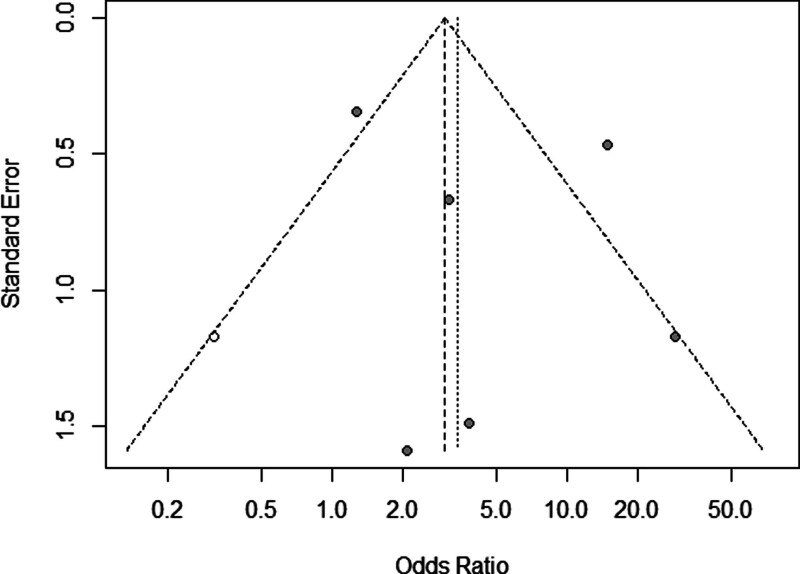
Funnel plot of the included studies.

A funnel plot was drawn for indicators that included more studies (incidence of severe illness in the vitamin D insufficiency group compared to that in the normal vitamin D level group). The results demonstrated symmetrical distribution of each study point to the left and right (Fig. 5), However, it is worth noting that the number of included studies is relatively small, and publication bias may still exist.

## 4. Discussion

Vitamin D is a group of fat-soluble prohormones composed of 5 different vitamins, the most important of which are vitamin D2 and D3. Calcitriol, the active form of vitamin D, stimulates innate immune responses by enhancing the chemotactic and phagocytotic responses of macrophages, as well as the production of antimicrobial peptides. Further, it strongly enhances the production of IL-10 by stimulating T regulatory cells and inhibiting Th1 and Th17 cell differentiation.^[19]^ Moreover, vitamin D supplementation has been proven to prevent respiratory infections. A study on 1300 healthy children and adolescents aged 3 to 17 years lasting 8 months showed that vitamin D supplementation moderately reduced non-influenza respiratory virus infections.^[20]^ Meanwhile, a high-quality meta-analysis of 11,321 participants showed a 12% overall protective effect of vitamin D supplementation against acute bacterial and viral respiratory infections among all participants (adjusted OR, 0.88; 95% CI, 0.81–0.96; *P* for heterogeneity < .001).^[21]^ In a study on 244 infants who completed follow-up from birth to 6 months of age, supplemental vitamin D doses of 400 to 600 IU/d were associated with lower risks of hospitalization and respiratory infection in the first 6 months of life compared with no supplementation.^[22]^

Vitamin D is commonly associated with calcium absorption and bone health. Moreover, over the past decade, it has been reported to have a key role in inflammatory and immune regulation. Despite this critical role, the prevalence of vitamin D deficiency remains widespread. Studies have found a significant association between 25(OH)D concentration and COVID-19 severity and mortality.^[23]^ Studies have shown that the severe acute respiratory syndrome coronavirus (SARS-CoV)-2 infects and activates macrophages through angiotensin-converting enzyme (ACE) 2 receptors, leading them to secrete less interferon and more cytokines and chemokines, including IL-1β and IL-6. Accumulated mononuclear macrophages produce several inflammatory cytokines, including TNF, IL-6, IL-1β, and so on, thus increasing disease severity.^[24]^ Vitamin D has been reported to inhibit the expression of renin, ACE, and angiotensin Ⅱ, and increase the concentration of ACE 2.^[25]^

Patients with COVID-19 demonstrating vitamin D insufficiency or deficiency, especially severe deficiency, are at an increased risk of progressing to severe status. Moreover, participants with poor vitamin D status seemingly have a higher risk of SARS-CoV-2- and COVID-19-related hospitalizations, as indicated in a previous meta-analysis.^[26]^ A causal relationship cannot be confirmed at this stage, as it is not possible to determine whether severe illness leads to a decline in vitamin D levels or vice versa. A study published by Zurita-Cruz showed that vitamin D supplementation reduced the risk of COVID-19 progression and death.^[27]^

While the current evidence overwhelmingly supports the hypothesis that vitamin D affects COVID-19 outcomes, this study has some limitations: Correlation does not equal causation. None of the literature included in the analysis addressed whether low vitamin D levels caused severe COVID-19 or severe COVID-19 caused low vitamin D levels. Therefore, these results should be interpreted with caution, as the cause and effect of these 2 factors remains uncertain. Study heterogeneity was significant. Due to the small number of studies on each key outcome indicator, the source of heterogeneity was not explored. Subgroup analysis was originally intended to be conducted by country; however, it was not performed as there were too few studies and the included countries were scattered. Therefore, only a random effects model was used to solve the heterogeneity of the study, which may affect the strength and extrapolation of study findings. The standards of vitamin D deficiency in children of different ages may be inconsistent; however, the included literature was not analyzed by age, so the relevant subgroup analysis was not presented in this paper. Therefore, the results of this study should be treated with caution. All included studies are small studies with dozens to hundreds of participants, and there is a lack of large multi-center RCT studies, so the results are not persuasive.

Although it is uncertain whether low levels of vitamin D are the cause or result of severe COVID-19, until effective childhood vaccines and specific medicines are widely available, adequate vitamin D supplementation may currently be the most cost-effective and safe way to treat and prevent COVID-19 in children. However, current clinical studies have not identified the optimal supplemental dose of vitamin D for the prevention of severe COVID-19 in a wide range of people, which needs to be explored in further basic and clinical trials.

## Acknowledgments

We would like to thank Editage [www.editage.cn] for English language editing.

## Author contributions

**Data curation:** Ji-Gan Wang.

**Formal analysis:** Ji-Gan Wang, Qiong-You Liang.

**Methodology:** Qiong-You Liang.

**Software:** Hui-Hong Dou.

**Validation:** Hui-Hong Dou.

**Writing – review & editing:** Hui-Hong Dou.

## Supplementary Material


