# Supplementary material for: Vitamin D levels in children and adolescents are associated with coronavirus disease-2019 outcomes: A systematic review and meta-analysis
Source: Medicine (Baltimore). 2024 Nov 1;103(44):e40245. doi: 10.1097/MD.0000000000040245 (PMC11537629; doi:10.1097/MD.0000000000040245)
Supplement: Supplementary file 1 [file medi-103-e40245-s001.pdf]

Characteristics of included studies

Study	Number of participants	Age						
Yılmaz K 2020	85	3M-18Y		Covid-19	Control group		Vitamin D Insufficient	Vitamin D normal group
			Total number of people	40	45	Total number of people	29	11
			Insufficient	11	3	Asymptomatic	3	5
			Deficiency	18	8	Mild	17	4
						Moderate	7	2
						Severe	2	0
Karakaya 2021	49	Median age 9Y		Deficiency 26	Insufficient 14			
Bayramoğlu E2021	103	12.2±4.92 Y		Deficiency	Insufficient		Vitamin D Insufficient	Vitamin D normal group
				43	41	Total number of people	84	19
						Asymptomatic	22	7
						Mild	31	9
						Moderate		
						Severe	31	3
Alpcan A2021	155	10.7±5.5 Y		Covid-1	Control		Vitamin D	Vitamin D normal

				9	group		Insufficient	group
			Total number of people	75	80	Total number of people	63	12
			Normal	12	27	Severe	8	0
			Insufficient	30	39			
			Deficiency	33	14			
Torpoco2022	31	Median age 8Y（1-13Y）					Vitamin D Insufficient	Vitamin D normal group
						Total number of people	10	21
						Severe	9	5
Zeidan 2022	380	Median age10.7Y	Total number of people	Covid-19	Control group		Vitamin D Insufficient	Vitamin D normal group
				180	200	Total number of people	94	86
			Normal	86	156	Moderate	68	66
			Insufficient	74	30	Severe	17	13
			Deficiency	20	14	Critical	9	7
Heidar 2022	44	range: 1-16Y					Vitamin D Insufficient	Vitamin D normal group
						Total number of	73	71

						people		
						Mild	4	39
						Moderate	24	25
						Severe	45	7
Peng D2022	116	Median (IQR) 7.8Y					Vitamin D Insufficient	Vitamin D normal group
						Total number of people	80	36
						Asymptomatic	3	4
Doğan 2022	196	8.9 ± 5.01 Y		Covid-19	Control group			
			Total number of people	88	88			
			Normal	1	21			
			Insufficient	52	50			
			Deficiency	35	17			

The Newcastle-Ottawa Scale (NOS) for Assessing the Quality of Studies Included into Present Meta-Analyses

The Newcastle-Ottawa Scale (NOS)

We downloaded the following scale from: http://www.ohri.ca/programs/ clinical_ epidemiology /oxford.asp, to evaluate the included studies qualities. The studies that met at least five NOS criteria were considered to be high quality studies.

1. **Newcastle-Ottawa Quality Assessment Scale: Case Control/Cross- Sectional Studies**

Note: A study can be awarded a maximum of one star for each numbered item within the Selection and Exposure categories. A maximum of two stars can be given for Comparability.

Selection

1) Is the case definition adequate?

a) yes, with independent validation ✹

b) yes, eg record linkage or based on self reports

c) no description

2) Representativeness of the cases

a) consecutive or obviously representative series of cases ✹□

b) potential for selection biases or not stated

3) Selection of Controls

a) community controls ✹

b) hospital controls

c) no description

4) Definition of Controls

a) no history of disease (endpoint) ✹

b) no description of source

Comparability

1) Comparability of cases and controls on the basis of the design or analysis

a) study controls for ________________ (Select the most important factor.)✵

b) study controls for any additional factor✵ (This criteria could be modified to indicate specific control for a second important factor.)

Exposure

1) Ascertainment of exposure

a) secure record (eg surgical records) ✵

b) structured interview where blind to case/control status ✵

c) interview not blinded to case/control status

d) written self report or medical record only

e) no description

2) Same method of ascertainment for cases and controls

a) yes ✵

b) no

3) Non-Response rate

a) same rate for both groups ✵

b) non respondents described

c) rate different and no designation

2. Newcastle-Ottawa Quality Assessment Scale: Cohort Studies

Note: A study can be awarded a maximum of one star for each numbered item within the Selection and Outcome categories. A maximum of two stars can be given for Comparability

Selection

1) Representativeness of the exposed cohort

a) truly representative of the average _____(describe) in the community✵□

b) somewhat representative of the average ________in the community✵ □

c) selected group of users eg nurses, volunteers

d) no description of the derivation of the cohort

2) Selection of the non exposed cohort

a) drawn from the same community as the exposed cohort ✵

b) drawn from a different source

c) no description of the derivation of the non exposed cohort

3) Ascertainment of exposure

a) secure record (eg surgical records) ✵□

b) structured interview □

c) written self report

d) no description

4) Demonstration that outcome of interest was not present at start of study

a) yes ✵

b) no

Comparability

1) Comparability of cohorts on the basis of the design or analysis

a) study controls for _____________ (select the most important factor) ✵

b) study controls for any additional factor ✵ (This criteria could be modified to indicate specific control for a second important factor.)

Outcome

1) Assessment of outcome

a) independent blind assessment ✵

b) record linkage □

c) self report

d) no description

2) Was follow-up long enough for outcomes to occur

a) yes (select an adequate follow up period for outcome of interest) ✵

b) no

3) Adequacy of follow up of cohorts

a) complete follow up - all subjects accounted for ✵

b) subjects lost to follow up unlikely to introduce bias - small number lost - > ___ % (select an adequate %) follow up, or description provided of those

lost)✵

c) follow up rate < ____% (select an adequate %) and no description of those lost

d) no statement

The Detailed Assessment Process of Every Included Article

Table S1 The Detailed Assessment Process of Case-Control/ Cross-sectional Studies Included into Present Meta-analyses

	Selection 1)	Selection 2)	Selection 3)	Selection 4)	Comparability 1)	Exposure 1)	Exposure 2)	Exposure 3)	Score
Karakaya [9]2021	a)	a)	b)	b)	a)	b)	a)	b)	5
Bayramoğlu E[10]2021	a)	a)	b)	b)	a)	a)	b)	c)	4
Alpcan A[11]2021	a)	a)	a)	a)	b)	a)	a)	c)	7
Torpoco[12]2022	a)	a)	a)	b)	b)	b)	a)	a)	6
Heidar[14]2022	a)	a)	b)	b)	b)	a)	a)	c)	5
Peng D[15]2022	a)	a)	b)	a)	a)	a)	a)	c)	6

Table S2 The Detailed Assessment Process of Cohort Studies Included into Present Meta-analyses

	Selection 1)	Selection 2)	Selection 3)	Selection 4)	Comparability 1)	Exposure 1)	Exposure 2)	Exposure 3)	Score
Yılmaz K[8]2020	a)	a)	a)	a)	a) b)	a)	b)	b)	8
Zeidan[13]2022	a)	a)	a)	a)	a) b)	a)	b)	b)	8
Doğan[16]2022	b)	a)	a)	a)	a)	a)	c)	b)	7
